# Lupeol- and Cis-Vaccenic Acid-Containing Bioactive Fraction of *Mondia whitei* Induces Vacuolation-Associated Cytotoxicity Through Autophagy–Lysosome Perturbation and Caspase-Dependent Apoptosis in Human Colorectal Adenocarcinoma Cells

**DOI:** 10.3390/biom16081123

**Published:** 2026-08-01

**Authors:** Saheed O. Anifowose, Mobarak S. Al Mosallam, Eman Abdullah Bahattab, Fahad S. Alotaibi, Mohammad Nasser Alkhrayef, Kamoru A. Adedokun, Abdulmujib Gboyega Yusuf, Ahmad Rady, Mansour I. Almansour, Ibrahim O. Alanazi, Badr A. Al-Dahmash

**Affiliations:** 1 Zoology Department, College of Science, King Saud University, Riyadh 11451, Saudi Arabia; 444105896@student.ksu.edu.sa (S.O.A.);; 2 Healthy Aging Research Institute, Health Sector, King Abdulaziz City for Science and Technology, Riyadh 11442, Saudi Arabia; 3 Advanced Agricultural and Food Technologies Institute, King Abdulaziz City for Science and Technology, Riyadh 11442, Saudi Arabia; 4 Disability Research Institute, King Abdulaziz City for Science and Technology, Riyadh 11442, Saudi Arabia; mkhuryef@kacst.gov.sa; 5 Immunology Department, Roswell Park Comprehensive Cancer Center, Buffalo, NY 14263, USA; kamoru.adedokun@roswellpark.org; 6 Graduate Division, University at Buffalo, Buffalo, NY 14263, USA; 7 Plant Protection Department, College of Food and Agricultural Sciences, King Saud University, Riyadh 11451, Saudi Arabia

**Keywords:** autophagy/lysosome perturbation, intrinsic apoptosis, bioactivity-guided fractionation, *Mondia whitei*, white ginger, lupeol and cis-vaccenic acid

## Abstract

Plant-derived natural products are essential and prominent contributors to drug discovery, especially as anticancer agents. Medicinal plants used in alternative therapy are often obscured by poor definitions of the underlying mechanisms behind their bioactivity. In this study, we investigated the cytotoxic and mechanistic effects of an enriched bioactive fraction derived from *Mondia whitei*. Bioactivity-guided fractionation was performed using C18 solid-phase extraction. The cytotoxic effects were evaluated using the MTT assay, while cellular morphology and mechanistic pathways were examined through microscopy, acridine orange staining, cathepsin-based assay, and immunoblotting of apoptosis- and autophagy-related proteins. Chemical profiling of the fraction was conducted using GC–MS analysis. The enriched fraction exhibited enhanced cytotoxicity at low microgram concentrations. Morphological assessment revealed prominent cytoplasmic vacuolation, while acridine orange staining indicated the accumulation of acidic vesicles. Cathepsin-based assays and immunoblot analysis of LC3-I/II confirmed lysosomal involvement and autophagy perturbation, whereas increased p62 levels suggested disruption of the autophagy–lysosome perturbation. In parallel, activation of intrinsic apoptosis was evidenced by the increased expression of caspase-9 and caspase-3. GC–MS profiling tentatively identified lupeol and cis-vaccenic acid as the major constituents of the fraction. The results from these studies demonstrate that the enriched fraction of *Mondia whitei* induced vacuolation-associated cytotoxicity through autophagy–lysosome perturbation and caspase-dependent apoptosis, providing mechanistic insight into its anticancer potential.

## 1. Introduction

The use of medicinal plants have long been documented for therapeutic purposes in the treatment of various diseases [1]. Phytochemicals represent a vast and structurally diverse group of natural products with distinct biological activities and modes of action [2]. White ginger *(Mondia whitei;* hereafter referred to as MW) is a medicinal plant widely used in African ethnopharmacology [3]. This plant has been reported to possess antioxidant, antimicrobial, and aphrodisiac properties [4,5]. Phytochemical investigations of MW have identified diverse classes of secondary metabolites [6], and more recently, its fractions have demonstrated promising anticancer potential [7]. The current body of scientific evidence on MW has linked chemical profiling with in silico molecular docking analyses to predict potential molecular targets of its bioactive constituents. Previous studies have identified cathepsins among the putative molecular targets of MW phyto-constituents [7]. The available reports have provided valuable insight into the phytochemical composition and potential biological targets of the plant; however, they are largely limited to cytotoxicity evaluation and computational predictions. The underlying cellular mechanisms remain experimentally unvalidated. Cathepsin, a key lysosomal enzyme, has been established for its roles in lysosomal function and the regulation of both autophagy and apoptosis [8]. On this pedestal, these findings provided a biological rationale for investigating autophagy–lysosome-associated responses and apoptotic signaling pathways to elucidate the mechanism of action of MW.

The involvement of key regulated cell death pathways, particularly apoptosis and autophagy–lysosome signaling, has not been experimentally established for MW. Addressing these gaps is essential for linking the phytochemical composition of MW to its biological activity. This approach will advance the current state of knowledge about MW from predictive observations toward a mechanistic understanding of its anticancer potential. Interestingly, small molecules capable of inducing apoptosis and regulated autophagic responses are central to anticancer drug discovery [9]. Apoptosis and autophagy are two fundamental cellular processes that help maintain homeostasis and regulate cellular responses to stress [10,11]. Apoptosis is a tightly regulated programmed cell death (PCD) and has long been a major focus of anticancer research because of its role in eliminating damaged or malignant cells [12]. Autophagy is a cellular recycling pathway that degrades and repurposes intracellular components to sustain cell survival under adverse conditions [13]. However, the role of autophagy in cancer is complex and highly context-dependent [14]. Depending on the cellular environment and disease stage, autophagy may either suppress tumor development by promoting the removal of damaged cellular components or support cancer cell survival under therapeutic stress [15]. Furthermore, there are reports on the extensive crosstalk between autophagy and apoptosis [16].

The elucidation of how bioactive natural products influence apoptosis and autophagy–lysosome signaling can provide valuable insight into their mechanisms of action and potential therapeutic applications in cancer. In this study, we employed a bioactivity-guided fractionation approach to identify a potent fraction from MW and systematically investigate its anticancer mechanism of action in colon adenocarcinoma cells. Bioactivity-guided fractionation is a widely used strategy in natural product research; this approach combines biological screening with sequential chemical separation. The enriched fractions thereby contain pharmacologically active constituents while reducing the complexity of crude extracts. Prioritizing fractions according to their biological activity rather than their chemical composition facilitates the identification of fractions with enhanced bioactivity and provides a focused starting point for mechanistic investigations. Consequently, this has become an important tool for linking phytochemical composition with biological function and for prioritizing fractions for downstream chemical characterization and pharmacological evaluation [17]. In this study, we employed a bioactivity-guided fractionation approach to isolate a potent fraction from MW and systematically investigated its anticancer mechanism of action in colon adenocarcinoma cells.

## 2. Materials and Methods

### 2.1. Plant Material and Extraction

The root of MW was collected from Iwo, Osun State, Nigeria, authenticated, and deposited at the Herbarium of the University of Ibadan under voucher number UIH-23782. The roots were air-dried, ground into a fine powder, and extracted by maceration in HPLC-grade methanol (Sigma-Aldrich, St. Louis, MO, USA) for 24 h at a 10% (*w*/*v*) ratio under continuous shaking at room temperature. The extract was filtered and concentrated under reduced pressure using a rotary evaporator at 40 °C to obtain the crude extract.

### 2.2. Solid-Phase Extraction (SPE) Fractionation

Bioactivity-guided fractionation of the crude extract of MW was performed using reversed-phase C18 solid-phase extraction (SPE) cartridges (Selton Scientific, Gujarat, India). C18 SPE was employed to enrich moderate polar to non-polar phytochemicals, including lipophilic secondary metabolites. This strategy helps in reducing the abundance of highly polar constituents and facilitates the concentration of bioactive metabolites for subsequent biological evaluation [18]. Prior to sample loading, SPE cartridges were conditioned with 5 mL of methanol, followed by equilibration with 5 mL of deionized water to activate the C18 cartridge. The crude extract was diluted in a methanol–water mixture (90:10, *v*/*v*) to ensure adequate solubility and compatibility with the SPE system. The prepared sample was then loaded onto the pre-conditioned C18 cartridge slowly to allow for optimal interaction with the stationary phase. The cartridge was thereafter flushed with 5 mL of water to remove unbound metabolites/very polar compounds. Eluents of increasing methanol percentages (20%, 40%, 60%, 80% and 100% methanol in water) were employed for the fractionation. Each fraction was collected separately, concentrated under reduced pressure to remove solvent, and reconstituted in methanol for subsequent biological assays. Collected fractions were designated according to their elution solvent composition using the format MW-SPE-C18–MeOH%, corresponding to the percentage of methanol used during elution. All collected fractions were subjected to thin-layer chromatography (TLC) to compare their phytochemical profiles. Based on similarities in their TLC profiles and preliminary bioactivity screening, the MW-SPE-C18-40% and MW-SPE-C18-60% fractions were pooled. Following fractionation, all collected fractions were screened for cytotoxic activity against Caco-2 colon adenocarcinoma cells using the MTT assay. Fraction selection for subsequent mechanistic investigations was guided by comparative cytotoxic potency, as determined by IC_50_ values. Comparative screening demonstrated that MW-SPE-C18-80% exhibited the greatest cytotoxic activity and the lowest IC_50_ value among all fractions tested. Consequently, MW-SPE-C18-80% was selected for all subsequent mechanistic and chemical profiling analyses.

### 2.3. Cell Culture and Viability Assay

Human adenocarcinoma cell lines included Caco-2 (ATCC HTB-37), HT-29 (ATCC HTB-38), A549 (ATCC, CRM-CCL-185) and a triple-negative breast adenocarcinoma cell line (MDA-mb 239; ATCC, CRM-HTB-26). The cell lines were originally obtained from the American Type Culture Collection (ATCC) and were available as frozen cells in our laboratory. Cells were cultured and maintained in Dulbecco’s Modified Eagle’s Medium (DMEM) supplemented with 10% fetal bovine serum (FBS) and 1% antibiotic–antimycotic solution (Gibco™, Grand Island, NY, USA) and incubated at 37 °C in a humidified atmosphere containing 5% CO_2_. Cells were sub-cultured at 80–90% confluence and used for experiments.

Cell viability was assessed using the MTT assay. Briefly, cells were seeded in 96-well plates at a density of approximately 5 × 10^4^ cells/mL. Serial dilutions of the test samples were then added to generate dose–response curves. Following 72 h of treatment, MTT reagent (0.5 mg/mL) was added to each well and incubated for 2 h. The resulting formazan crystals were dissolved in isopropanol, and absorbance was measured at 570 nm using a microplate reader. Half-maximal inhibitory concentration (IC_50_) values were calculated using nonlinear regression analysis in Origin software 10.3.0.197 (OriginLab Corporation, Northampton, MA, USA). Cytotoxicity against normal cells was carried out to assess cancer selectivity, and the cytotoxic effect of MW-SPE-C18-80% was evaluated in L-929 murine fibroblast cells (NCTC clone 929, ATCC CCL-1™) using the MTT assay under the same experimental conditions as those used for the cancer cell lines. Vinblastine sulfate (Sigma-Aldrich, St. Louis, MO, USA) was used as the positive control. Cell viability was determined after 72 h of treatment, and IC_50_ values were calculated as described above.

### 2.4. Morphological Assessment

Caco-2 cells were treated with the MW active fraction at 50 and 100 µg/mL, and methanol was used as the vehicle control. Cellular morphology was examined using brightfield microscopy on a Motic AE31E inverted microscope (Motic, Xiamen, China). Morphological changes, including cytoplasmic vacuolation and cell rounding, were documented at 24 h and 48 h post-treatment. Cells were stained with acridine orange (5 µg/mL) for 10 min at 37 °C, washed with phosphate-buffered saline (PBS) supplemented with 25 mM HEPES to maintain pH stability during imaging, and immediately visualized using an EVOS FL fluorescence microscope (Thermo Fisher Scientific, Waltham, MA, USA). This approach was employed to assess the formation of acidic vesicular organelles and cytoplasmic vacuolation. Lysosomal involvement was further evaluated using a fluorogenic cathepsin L assay kit (Magic Red; ab270774, Abcam, Cambridge, UK). Cells were incubated with the substrate for 30 min at 37 °C in the dark and subsequently imaged live using fluorescence microscopy. Changes in the fluorescence intensity and intracellular distribution were interpreted as indicators of lysosomal activity. The quantitative analysis of the representative micrographs were done using ImageJ 1.54g analysis software, and data were presented as bar charts with the statistical analysis.

### 2.5. Immunofluorescence for p62

Caco-2 cells were seeded on glass coverslips and allowed to reach approximately 70–80% confluence prior to treatment. Cells were treated with 50 and 100 µg/mL concentrations of MW for 24 h. Following treatment, cells were fixed with 3.75% paraformaldehyde for 15 min at room temperature and subsequently washed with phosphate-buffered saline (PBS). Fixed cells were permeabilized by quick-wash in 0.1% Triton X-100 in PBS. Cells were then incubated with anti-SQSTM1/p62 rabbit monoclonal antibody (ab240635, Abcam, Cambridge, UK) at 37 °C for 1 h. After washing with PBS, cells were incubated with Alexa Fluor 488-conjugated goat anti-rabbit secondary antibody for 1 h at 37 °C. Nuclei were counterstained with 4′,6-diamidino-2-phenylindole (DAPI). Coverslips were mounted onto glass slides using antifade mounting medium to minimize photobleaching. Fluorescence images were acquired using a Nikon Eclipse Ci fluorescence microscope equipped with an NIS-Elements imaging system (Nikon Corporation, Tokyo, Japan). The quantitative analysis of the representative micrographs was done using ImageJ analysis, and software and data were presented as bar charts with the statistical analysis.

### 2.6. Immunoblotting Assay of Autophagic and Apoptotic Proteins

Following treatment, cells were harvested and lysed using M-PER™ Mammalian Protein Extraction Reagent (Thermo Fisher Scientific, Waltham, MA, USA) supplemented with protease inhibitors. The extraction was performed on ice for 30 min with intermittent mixing and subsequently centrifuged at 14,000× *g* for 30 min at 4 °C. The lysate was quantified using the Bradford assay, and all samples were normalized to equal protein concentrations. An amount of 25 µg of the lysates was mixed with 2× Laemmli loading buffer and denatured at 95 °C for 5 min. SDS PAGE was performed on 12% polyacrylamide gel, which was subsequently transferred onto polyvinylidene difluoride (PVDF) membranes via wet transfer at 80–100 V for 1–2 h. Following transfer, membranes were blocked with 5% BSA prepared in Tris-buffered saline with 0.1% Tween-20 (TBST) for 1 h at room temperature.

Membranes were incubated overnight at 4 °C with primary antibodies diluted in blocking buffer, including anti-caspase-3 (ab13847), anti-caspase-9 (ab2013), anti-LC3A/B (ab128025), anti-SQSTM1/p62 (ab240635), and anti-GAPDH (1:2500) as the loading control. After primary antibody incubation, membranes were washed three times with TBST for 5–10 min each. Subsequently, membranes were incubated with horseradish peroxidase (HRP)-conjugated anti-rabbit secondary antibodies at room temperature. Membranes were washed again three times with TBST to remove excess secondary antibodies. Protein bands were detected using an enhanced chemiluminescence (ECL) substrate and visualized using a chemiluminescent imaging system. Densitometric analysis was performed using ImageJ software (version 1.54g), and protein expression levels were normalized to GAPDH.

### 2.7. GC–MS Analysis

GC–MS analysis of the enriched bioactive fraction was performed on an Agilent 5977B GC system coupled to a mass selective detector (Agilent Technologies, Santa Clara, CA, USA). Compound separation was done with an HP-5MS Ultra Inert capillary column (30 m × 0.25 mm internal diameter; 0.25 µm film thickness), while helium served as the carrier gas at a constant flow rate of approximately 1.0 mL/min. The oven temperature parameter was set at 80 °C with a 2 min hold at the beginning and then gradually increased to 300 °C to facilitate the elution of compounds of higher molecular weight, which resulted in a total run time of 60 min. Mass spectra were acquired under electron ionization conditions at 70 eV, with the ion source, and quadrupole temperatures were maintained at 230 °C and 150 °C. A solvent delay of 7 min was applied, and spectral data were collected over a mass-to-charge (*m*/*z*) range of 40–550. Data acquisition and processing were performed using Agilent MassHunter software (version 10.0). Compound identification was achieved by matching the obtained spectra against entries in the NIST 14.L mass spectral library.

### 2.8. Statistical Analysis

All experiments were performed in at least three independent replicates, and data are presented as mean ± standard deviation (SD). Statistical analyses were conducted using one-way analysis of variance (ANOVA), followed by Tukey’s post hoc test for multiple comparisons using SPSS software (version 20; IBM Corp., Armonk, NY, USA). Statistical significance was defined as *p* < 0.05, *p* < 0.01, and *p* < 0.001.

IC_50_ values were determined by nonlinear regression using Origin software (OriginPro 2026 (64-bit) SR1 10.3.0.197 OriginLab Corporation, Northampton, MA, USA). This study utilized OpenAI for language polishing and grammatical editing to enhance the clarity and readability of the manuscript.

## 3. Results

### 3.1. Bioactivity-Guided SPE Fractionation Enriched Cytotoxic Activity of Mondia whitei

**Figure 1:**
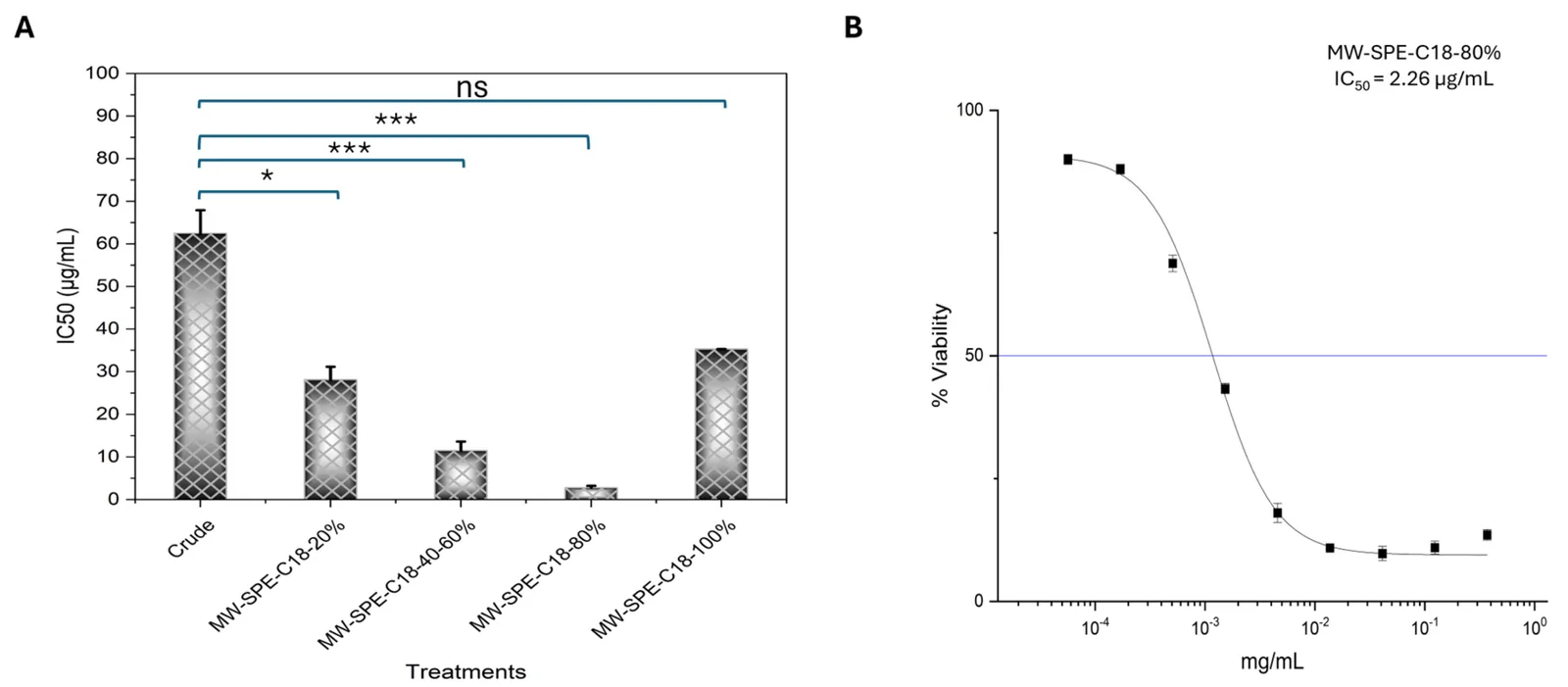
Cytotoxic activity and dose–response profile of *Mondia whitei* SPE fractions in Caco-2 cells. (**A**) IC_50_ values of crude extract and C18 SPE-derived fractions of MW determined using MTT assay in Caco-2 cells. Data are presented as mean ± SD of three independent experiments, and statistical significances are denoted as * *p* < 0.05, and *** *p* < 0.001; ns: not significant; (**B**) dose–response curve of most active fraction (MW-SPE-C18-80%) showing cell viability percentage as function of concentration.

The cytotoxic effects of the crude extract and C18 SPE-derived fractions of MW were evaluated in Caco-2 using the MTT assay (Figure 1A). The crude extract exhibited moderate activity with an IC_50_ of 62.49 ± 5.42 µg/mL. Stepwise elution resulted in a polarity-dependent enhancement of cytotoxicity. The 20% methanol fraction showed improved activity (28.11 ± 3.04 µg/mL, *p* < 0.05 vs. crude). A further increase in potency was observed for the pooled 40–60% fraction (11.50 ± 2.11 µg/mL, *p* < 0.001). Interestingly, the 80% fraction displayed the highest cytotoxic activity (2.26 ± 0.43 µg/mL, *p* < 0.001), representing a ~20-fold increase in potency relative to the crude extract. In contrast, the 100% methanol fraction exhibited reduced activity (35.27 ± 0.04 µg/mL) and was not significantly different from the crude extract (ns). Figure 1B shows a representative dose–response curve of the MW-SPE-C18-80% fraction against Caco-2 cells. The steep decline in cell viability across the mid-concentration range indicates strong, dose-dependent cytotoxicity, with near-complete loss of viability at higher concentrations. Further bioactivity testing was performed on the most active fraction (MW-SPE-C18-80%) against a panel of cancer cell lines, including HT-29, A549 and MDA-MB 231 cell lines, using the MTT assay (Table 1).

More interestingly, the cytotoxic effect of MW-SPE-C18-80% was further assessed in the non-cancerous L-929 fibroblast cell line to evaluate cancer selectivity. The fraction exhibited an IC_50_ of 23.72 µg/mL. This is approximately 10.5-fold higher than that observed in Caco-2 cells, corresponding to a selectivity index of 10.49. These findings indicate preferential cytotoxicity toward colorectal cancer cells. In addition, vinblastine sulfate, which was included as the positive control, exhibited an IC_50_ value of 0.124 µg/mL against Caco-2 cells. Although MW-SPE-C18-80% was less potent than vinblastine, it demonstrated marked cytotoxic activity (IC_50_ = 2.26 ± 0.43 µg/mL) together with preferential toxicity toward cancer cells compared with normal L-929 fibroblasts (IC_50_ = 23.72 µg/mL). The dose–response curve for the viability assay is presented in the Supplementary Materials.

### 3.2. MW-SPE-C18-80% Induced Vacuolation and Lysosome-Associated Cellular Changes

**Figure 2:**
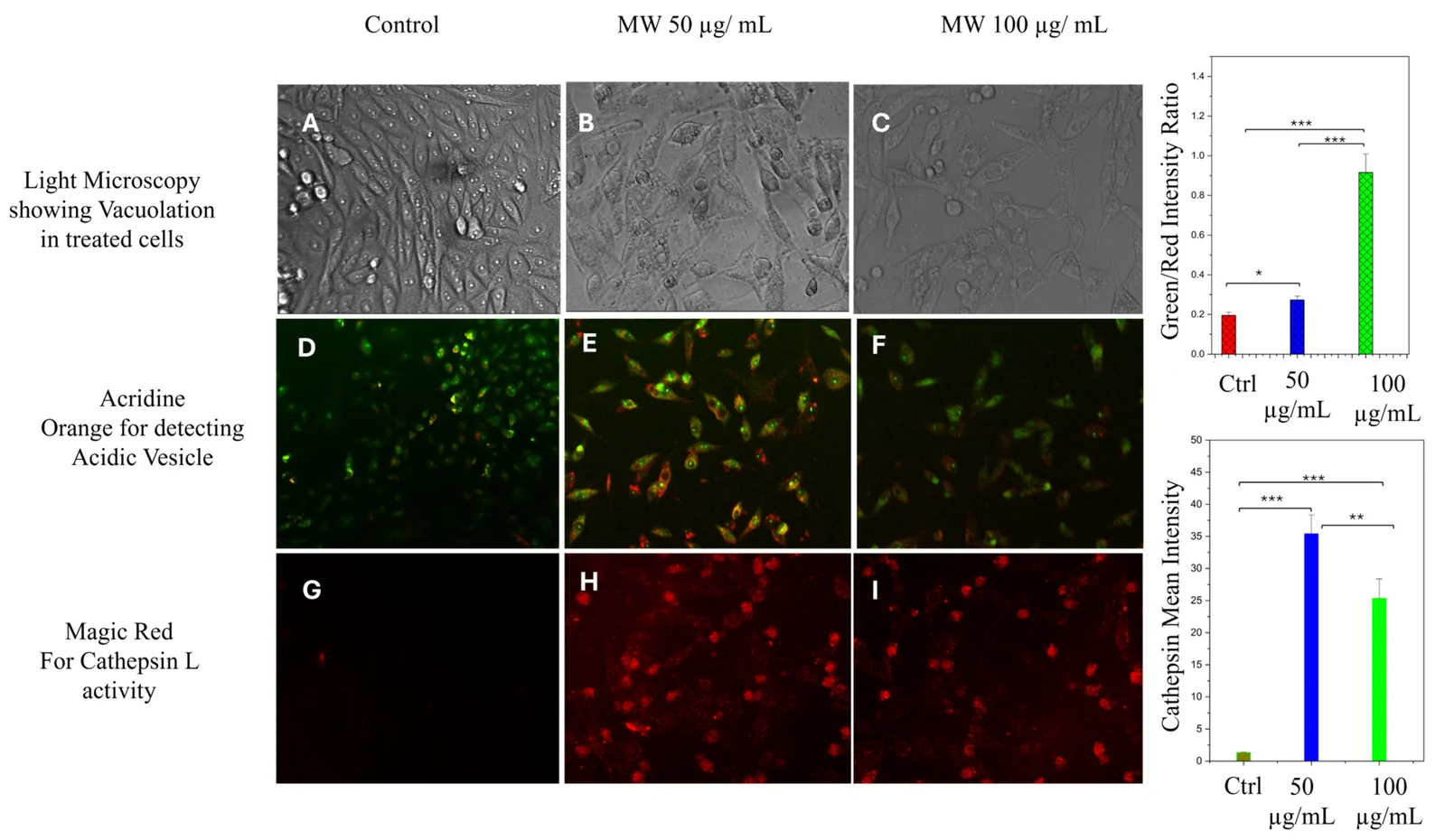
Vacuolation induction and lysosomal involvement by MW-SPE-C18-80% bioactive fraction. (**A**–**C**) Representative brightfield photomicrographs showing cellular morphology untreated cells and cells treated with MW-SPE-C18-80% (50 and 100 µg/mL) for 24 h. (**D**–**F**) Acridine orange staining of acidic vesicular compartments and (**G**–**I**) Magic Red™ cathepsin L staining. Quantitative analysis of acridine orange green/red fluorescence ratio and cathepsin L fluorescence intensity is presented alongside representative images. Data are expressed as mean ± SD from three independent experiments. Statistical significances denoted as * *p* < 0.05, ** *p* < 0.01, and *** *p* < 0.001.

The bioactive fraction MW-SPE-C18-80% produced pronounced, dose-dependent morphological alterations in Caco2 cells. As shown in Figure 2A–C, control cells (photomicrograph A) displayed typical epithelial morphology with minimal cytoplasmic vacuolation. In contrast, cells treated with 50 µg/mL (photomicrograph B) exhibited evident intracellular vacuole formation, while treatment at 100 µg/mL (photomicrograph C) led to extensive vacuolation accompanied by cell rounding and structural disintegration, indicative of progressive cellular stress. To further characterize the nature of the observed vacuolation, acridine orange (AO) staining was employed to detect acidic vesicular compartments. Control cells (Figure 2, photomicrograph D) showed predominantly green fluorescence, consistent with viable cells and minimal acidic vesicle accumulation. In treated cells, a marked increase in red/orange fluorescence was observed at 50 µg/mL (photomicrograph E) and became more pronounced at 100 µg/mL (photomicrograph F), indicating the increased formation of acidic vesicular organelles. These findings suggest that the vacuolation phenotype is associated with enhanced vesicular acidification. The green/red fluorescence ratio of the treated caco2 cells (Figure 2) shows dose-dependent effects, which are statistically significant compare to the untreated cells, supporting the increased formation of acidic vesicular organelles.

To assess lysosomal involvement, the cathepsin L activity was evaluated using a fluorogenic substrate. Control cells (Figure 2, photomicrograph G) exhibited minimal fluorescence, reflecting low basal lysosomal activity. In contrast, treated cells displayed a substantial increase in punctate red fluorescence at both 50 µg/mL (photomicrograph H) and 100 µg/mL (photomicrograph I), indicating enhanced cathepsin-associated lysosomal signaling. The fluorescence pattern was characterized by discrete puncta distributed throughout the cytoplasm, consistent with lysosomal localization. Altogether, these observations demonstrate that MW-SPE-C18-80% induces vacuolation accompanied by an increased accumulation of acidic vesicles and enhanced lysosomal activity, supporting the involvement of autophagy–lysosome perturbation in the observed cellular response.

### 3.3. MW-SPE-C18-80% Perturbs Autophagy–Lysosome Perturbation and Promotes p62 Accumulation

**Figure 3:**
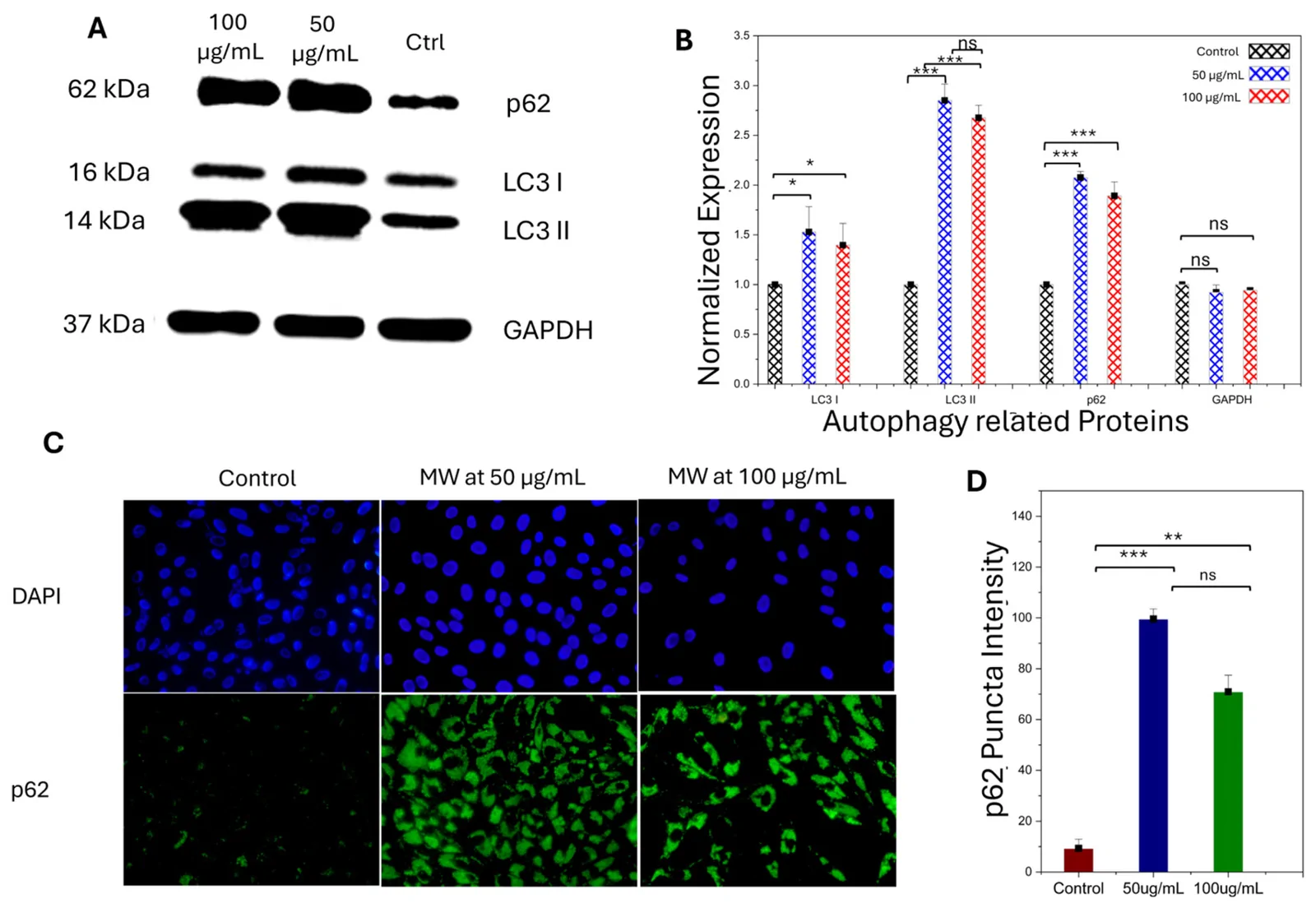
Bioactive fraction induces accumulation of autophagy-related proteins and p62 puncta increase structures. (**A**) Western blot showing LC3-I/II, p62, and GAPDH expression in untreated cells and cells treated with MW-SPE-C18-80% (50 and 100 µg/mL) for 24 h. (**B**) Densitometric analysis of LC3-I, LC3-II, and p62 protein expression normalized to GAPDH. Data is presented as mean ± SD from 3 independent experiments (*n* = 3). Picture panels in (**C**) show immunofluorescence images of p62 (green) and nuclei stained with DAPI (blue) in untreated and treated cells. (**D**) Quantification of p62 puncta. Data are presented as mean ± SD and n = 3. Statistical significance was determined using one-way ANOVA followed by Tukey’s multiple comparisons test (* *p* < 0.05, ** *p* < 0.01, *** *p* < 0.001, ns: not significant).

To investigate the involvement of autophagy-related processes, the expression and localization of LC3 and p62 were assessed using Western blotting and immunofluorescence analysis. Western blot analysis revealed that treatment with the bioactive fraction resulted in a marked increase in LC3-II levels compared to control cells (Figure 3A). Densitometric quantification confirmed that LC3-II expression was significantly elevated in both treatment groups (*p* < 0.001) compared to the control. No significant difference was observed between 50 and 100 µg/mL (Figure 3B). LC3-I and II exhibited a modest but significant increase relative to the control without a dose-dependent pattern.

Concurrently, p62 protein levels were significantly increased in treated cells compared to the control (*p* < 0.001), with no significant difference between the treatment concentrations, suggesting a plateau response. To further validate these findings, p62 localization was examined by immunofluorescence. Control cells displayed weak and diffuse p62 staining, whereas treated cells exhibited a marked increase in p62-associated fluorescence characterized by prominent punctate structures (Figure 3C), indicative of accumulation of autophagy-associated cargo. Importantly, the concurrent increase in LC3-II and p62, together with the formation of p62-positive puncta, suggests impaired autophagic turnover rather than activated autophagic flux. These findings support the notion that MW-SPE-C18-80% induces perturbation of the autophagy–lysosome pathway, leading to accumulation of autophagy-related structures.

### 3.4. Activation of Intrinsic Apoptotic Signaling by MW-SPE-C18-80%

**Figure 4:**
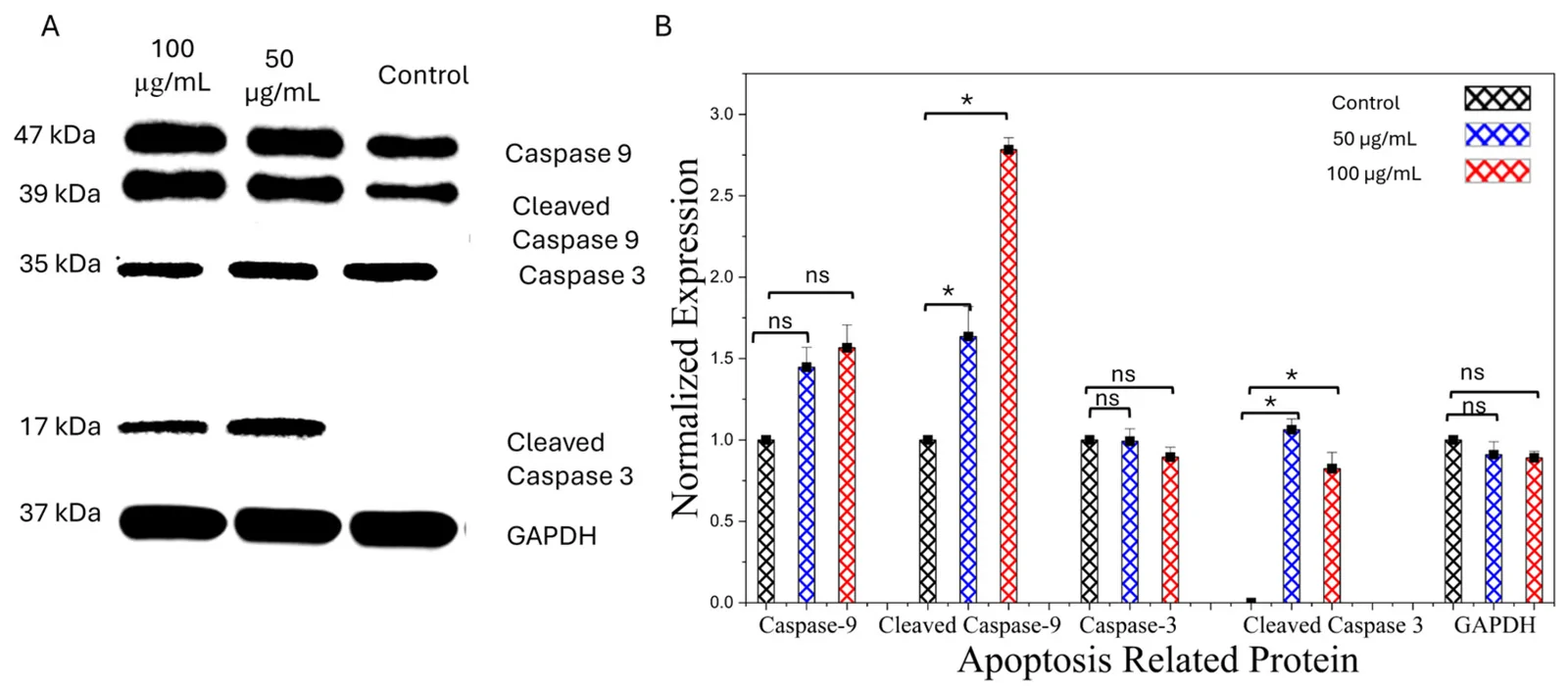
Activation of caspase-mediated apoptosis in treated cells. (**A**) Western blot analysis of caspase-9, cleaved caspase-9, caspase-3, and cleaved caspase-3 in control and treated cells. GAPDH was used as loading control. (**B**) Quantitative analysis of protein expression normalized to GAPDH (n = 3). Statistical significances denoted as * *p* < 0.05 and ns: not significant.

Western blot analysis was performed to evaluate the activation of caspase-mediated apoptotic pathways following treatment. As shown in Figure 4A, treatment resulted in a marked increase in the cleavage of both caspase-9 and caspase-3 compared to the control. Full-length caspase-9 exhibited a modest, non-significant increase in treated groups relative to the control. In contrast, the cleaved caspase-9 levels were significantly elevated in the treated samples, with a more pronounced increase observed at the higher concentration. Quantitative analysis (Figure 4B) confirmed that the cleaved caspase-9 expression was significantly higher in treated groups compared to the control (* *p* < 0.05), indicating activation of the intrinsic apoptotic pathway.

Similarly, the full-length caspase-3 levels did not differ significantly between the control and treated groups. However, cleaved caspase-3 was strongly induced following treatment, with minimal or no detectable signal in the control samples. Statistical analysis demonstrated a significant increase in the cleaved caspase-3 levels in the treated groups compared to the control (* *p* < 0.05), confirming activation of downstream apoptotic execution mechanisms. Notably, the reduction in full-length caspase-3 observed in the treated samples is consistent with proteolytic processing into its active cleaved form. Our findings indicate that the bioactive fraction induces apoptosis via activation of the intrinsic caspase cascade, involving upstream caspase-9 activation and downstream caspase-3 cleavage.

### 3.5. GC–MS Profiling Identifies Major Bioactive Constituents in MW-SPE-C18-80%

**Figure 5:**
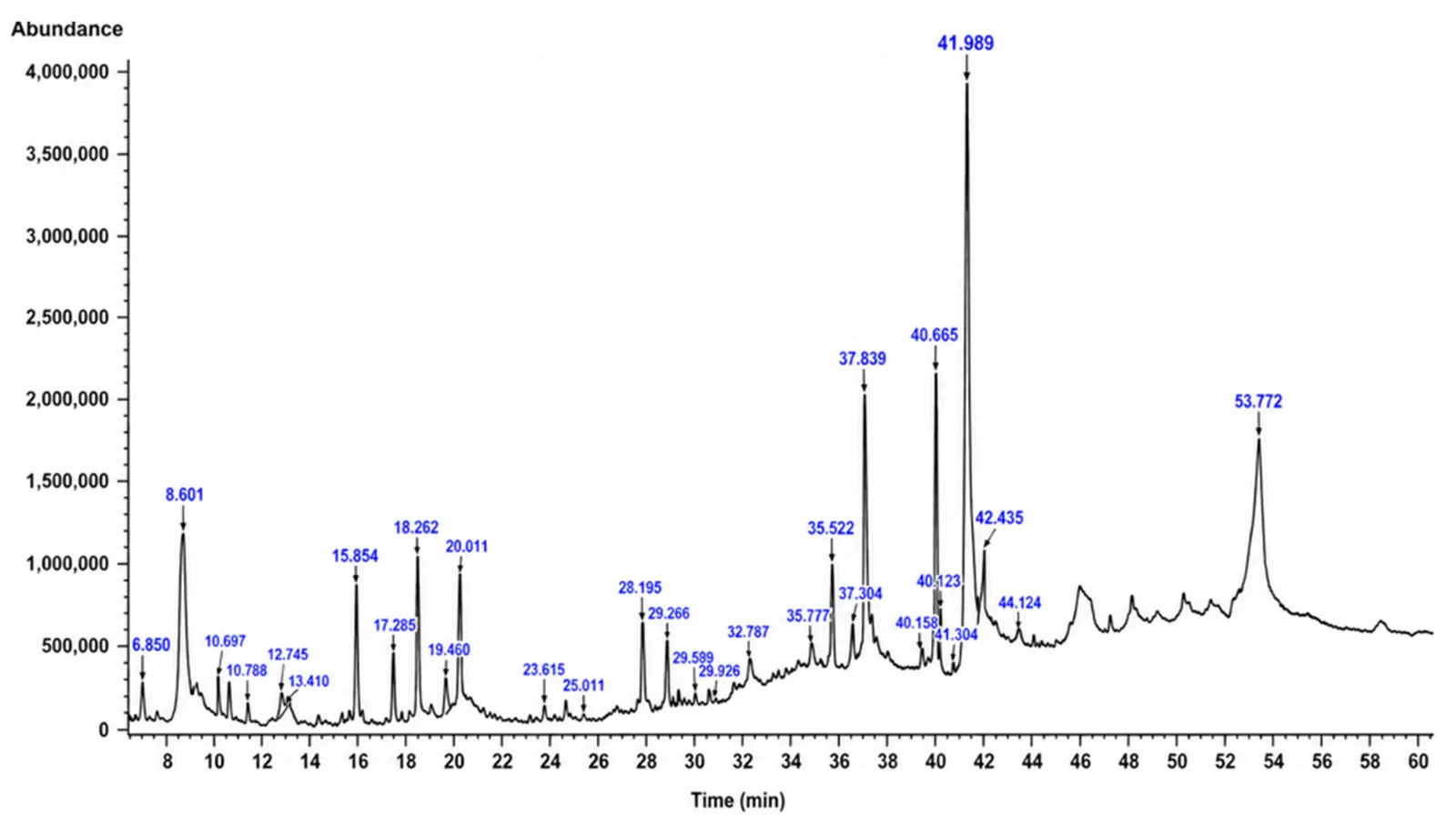
GC–MS chromatogram of bioactive fraction of MW.

Gas chromatography–mass spectrometry (GC–MS) analysis was performed to characterize the chemical composition of the bioactive fraction. The chromatographic profile revealed a complex mixture of predominantly lipid-derived compounds and terpenoids, with a total of 35 detectable peaks (Figure 5). Among the identified compounds, cis-vaccenic acid (Rt = 41.989 min) was the most abundant constituent, accounting for 28.46% of the total peak area (Table 2). This was followed by the triterpenoid lupeol (Rt = 53.772 min), which contributed 24.85% of the total composition. Other notable components included n-hexadecanoic acid (8.04%), benzyl nitrile (9.60%), and methyl oleate (4.11%), along with several unsaturated aldehydes, such as 2,4-dodecadienal and 2-undecenal, present in lower proportions.

Overall, the chemical profile was dominated by fatty acids and their derivatives, which collectively represented a substantial proportion of the extract, alongside a smaller but significant contribution from terpenoid compounds. Minor constituents included sesquiterpenes (e.g., bisabolene epoxide and eudesmol derivatives) and reactive aldehydes, which are known to possess biological activity.

## 4. Discussion

In this study, we attempted to elucidate the mechanism of action of the bioactive fraction MW-SPE-C18-80%, derived from *Mondia whitei*. The fraction demonstrated enhanced cytotoxic activity compared to the crude extract. It induced a distinct vacuolation-associated phenotype in colon adenocarcinoma cells. Interestingly, MW-SPE-C18-80% displayed differential cytotoxicity across the tested cancer cell lines. Caco-2 cells showed the greatest sensitivity and MDA-MB-231 cells demonstrated minimal response. This may possibly reflect differences in cellular metabolism, apoptotic competence, and autophagy–lysosome signaling [29]. The examination of cytotoxicity with L-929 fibroblasts enabled a preliminary assessment of cancer selectivity [30]. The lower sensitivity of L-929 cells compared with Caco-2 cells suggests that MW-SPE-C18-80% exhibits preferential selectivity toward colon cancer cells. Comprehensive evaluation of the bioactive fraction through additional normal human cell lines and in vivo models is required to establish its safety profile and therapeutic window. The bioactive fraction exhibited lower potency compared to the vinblastine sulfate (reference chemotherapeutic agent). The pronounced cancer selectivity (SI ≈ 10.5) exhibited by the enriched fraction indicates that the fraction contains bioactive constituents with promising anticancer potential. This fraction warrants further purification and characterization. Meanwhile, the available reports on MW have largely focused on its phytochemical composition [6], antioxidant potential [31], and in silico prediction of molecular targets [7]. Experimental validation of its mechanism of cell death remains limited. Our approach therefore extends the existing knowledge by providing mechanistic insight into the cellular responses elicited by the bioactive fraction of this plant. A prominent feature observed in treated cells was the formation of extensive cytoplasmic vacuolation, accompanied by alterations in lysosomal-associated fluorescence. Such phenotypes are often associated with perturbations in vesicular trafficking and lysosomal function [32]. Consistent with this, the observed changes in cathepsin-associated signaling suggest involvement of lysosomal pathways [33], indicating that the vacuolation phenotype is not merely morphological but reflects underlying functional alterations in the autophagy–lysosome perturbation. This is in line with previous reports showing that lysosomal dysfunction can act as a central mediator of stress responses leading to cell death [34].

In this current mechanistic investigation of MW, our results revealed modulation of autophagy-related markers. Increased LC3-II levels and the concurrent accumulation of p62 in treated cells was evidenced in Caco2 cells treated with the bioactive fraction. LC3-II accumulation is commonly associated with autophagosome formation [35], which complements the observed vacuolations after treatment with the bioactive fraction, marking the involvement of the autophagy–lysosome pathway. However, the concomitant increase in p62 suggests altered autophagic turnover rather than efficient autophagic degradation [36]. This pattern is indicative of perturbation of the autophagy–lysosome axis, potentially reflecting impaired cargo clearance or dysregulated autophagic flux [37]. Similar observations have been reported in studies where cellular stress disrupted lysosomal processing and the accumulation of autophagy-related intermediates [38]. Subsequent mechanistic exploration of MW suggests the activation of the intrinsic apoptotic signaling pathway. The bioactive fraction induced cleavage of caspase-9 and caspase-3. The involvement of caspase-9 suggests activation of the intrinsic apoptotic pathway, which is typically associated with mitochondrial signaling [39]. Although the responses of autophagy-associated proteins and cleaved caspase-3 were not strictly linear between 50 and 100 µg/mL, the data demonstrate concentration-dependent modulation with evidence of a plateau at the higher concentration. This pattern may reflect progression from an early stress response toward a later stage of apoptosis, where severe cellular injury and commitment to cell death alter the dynamics of autophagy-associated proteins and executioner caspase activation [40]. Conversely, cleaved caspase-9 continued to increase at 100 µg/mL, supporting sustained activation of the intrinsic apoptotic pathway [41]. The coexistence of autophagy–lysosome perturbation and apoptosis supports the concept of crosstalk between these pathways [16]. Similar studies report dual apoptosis and autophagy modulation by medicinal plants to induce prolonged cellular stress or failure of adaptive autophagy that may culminate in apoptotic cell death [42]. Based on the evidence from these current studies, we propose this as a working mechanistic model rather than a demonstrated causal pathway. The present study does not establish the temporal or mechanistic relationship among these events, and therefore future studies employing selective pharmacological inhibitors or genetic approaches will be required to determine whether vacuolation, autophagy–lysosome perturbation, and apoptosis are causally linked or represent parallel cellular responses to treatment.

Moreover, the metabolite profiling of MW-SPE-C18-80% via GC-MS analysis tentatively suggests the presence of cis-vaccenic acid and lupeol as major constituents. Lupeol, a pentacyclic triterpenoid, has been widely reported to induce apoptosis and modulate cellular signaling pathways in various cancer models [43,44], including activation of caspase cascades [45]. Its presence in the fraction may therefore contribute to the observed apoptotic effects. Similarly, fatty acids such as cis-vaccenic acid have been implicated in modulating membrane dynamics and cellular stress responses [19]. However, the present study was conducted using a semi-purified fraction, and the observed biological effects likely reflect the combined action of multiple constituents rather than a single compound. Taken together with the results emanating from the current studies, our findings support a model in which MW-SPE-C18-80% induces cellular stress characterized by vacuolation and lysosomal involvement, leading to perturbation of the autophagy–lysosome pathway and the subsequent activation of intrinsic apoptotic signaling. This integrated response highlights the potential of MW as a source of compounds capable of modulating multiple cell death pathways. Despite these insights, several limitations are acknowledged. The precise molecular targets of the active constituents were not identified, and the contribution of individual compounds to the observed effects remains to be determined. Future studies should focus on the further purification and structural elucidation of the active components, as well as functional validation using pathway-specific inhibitors and mitochondrial assays to clarify the sequence of events leading to cell death. Additionally, in vivo studies will be required to evaluate the therapeutic potential and safety profile of these bioactive fractions.

## 5. Conclusions

This study demonstrates that a bioactive fraction derived from *Mondia whitei* exhibits enhanced cytotoxic activity compared to the crude extract. The enriched fraction induced a distinct vacuolation-associated phenotype in caco2 cells. Mechanistically, the fraction was associated with perturbation of the autophagy–lysosome pathway. This was evidenced by LC3 modulation, p62 accumulation, and altered cathepsin-associated signaling, alongside activation of intrinsic apoptotic signaling through caspase-9 and caspase-3 cleavage. These findings suggest that the cytotoxic effects of the fraction involve the coordinated disruption of cellular homeostasis and engagement of multiple cell death pathways. Chemical profiling identified lupeol and cis-vaccenic acid as major constituents, providing a plausible basis for the observed biological activity, although the individual contributions of these metabolites remain to be elucidated. Overall, this work provides experimental insight into the cellular mechanisms underlying the anticancer potential of *Mondia whitei* and highlights the importance of further studies to isolate active compounds and define their specific molecular targets.

## Figures and Tables

**Table 1:** Cytotoxicity of enriched MW-SPE-C18-80% fraction against panel of cancer cells.

Cell Line	Origin	IC_50_ (µg/mL) of MW-SPE-C18-80%	Selectivity Index (SI)
Caco2	Human colorectal adenocarcinoma	2.26 ± 0.43	10.49
HT-29	Human colon adenocarcinoma	5.17 ± 1.89	4.59
A549	Human lung adenocarcinoma	11.06 ± 2.29	2.14
Mda-mb 231	Human breast adenocarcinoma	NA	NA
L-929	Murine fibroblast (normal)	23.72	-

Note: Vinblastine was included as a positive control in the Caco-2 cytotoxicity assay and exhibited an IC_50_ of 0.124 µg/mL ± 0.08 µg/mL, confirming the responsiveness of the assay. NA: no activity; the IC_50_ value was above 100 µg/mL.

**Table 2:** GC–MS profiling of MW-SPE-C18-80% and putative chemical classes with associated bioactivity relevance.

RT (min)	Tentatively Identified Compound	Area (%)	Chemical Class	Putative Bioactivity	Ref.
41.989	cis-Vaccenic acid	28.46	Monounsaturated fatty acid	Membrane disruption, mitochondrial stress, apoptosis induction	[19]
53.772	Lupeol	24.85	Triterpenoid	Anticancer, apoptosis induction, autophagy–lysosome perturbation, caspase activation	[20]
37.839	*n*-Hexadecanoic acid	8.04	Saturated fatty acid	Pro-apoptotic, lipid-mediated cytotoxicity	[21]
40.665	9-Octadecenoic acid methyl ester	4.11	Fatty acid ester	Membrane interaction, lipid signaling	[22]
18.262	2,4-Dodecadienal	3.03	Unsaturated aldehyde	Reactive electrophile, oxidative stress, membrane damage	
15.854	2-Decenal (E)	2.18	Aldehyde	–	-
36.522	Oleic acid	1.87	Monounsaturated fatty acid	Modulates membrane fluidity and apoptosis	[23]
20.011	2-Undecenal	1.80	Aldehyde	–	-
42.435	cis-10-Nonadecenoic acid	0.84	Fatty acid	Adjunct/complementary anticancer effect	[24]
40.484	Linoleic acid methyl ester	0.50	Polyunsaturated fatty acid	Lipid signaling, ROS modulation	[25]
23.610	Fatty acid derivative (alkynyl ester)	0.30	Lipid derivative	Membrane perturbation	[26]
29.582	Eudesmol derivative	0.27	Sesquiterpene alcohol	Apoptosis induction	[27]
44.124	cis-13-Eicosenoic acid	0.25	Fatty acid	–	-
29.465	Bisabolene epoxide	0.20	Sesquiterpene	Antioxidant potential	[28]

Note: The compounds are ranked according to the relative peak area (%) to highlight the major tentatively identified constituents relevant to the biological activity discussed in this study. Reported biological activities were compiled from the literature and are intended to provide biological context; they do not constitute experimental validation in the present study.

## Data Availability

Data is contained in the article.

## Supplementary Materials

The following supporting information can be downloaded at: https://www.mdpi.com/article/10.3390/biom16081123/s1, Original Western Blots images.

## Abbreviations

The following abbreviations are used in this manuscript:

MW	*Mondia whitei*
SPE	Solid-phase extraction
TLC	Thin-layer chromatography
NA	No activity
