# Supplementary material for: Lupeol- and Cis-Vaccenic Acid-Containing Bioactive Fraction of Mondia whitei Induces Vacuolation-Associated Cytotoxicity Through Autophagy–Lysosome Perturbation and Caspase-Dependent Apoptosis in Human Colorectal Adenocarcinoma Cells
Source: Biomolecules. 2026 Aug 1;16(8):1123. doi: 10.3390/biom16081123 (PMC13510442; doi:10.3390/biom16081123)
Supplement: Supplementary file 1 [file biomolecules-16-01123-s001.zip › biomolecules-4363392-supplementary.pdf]

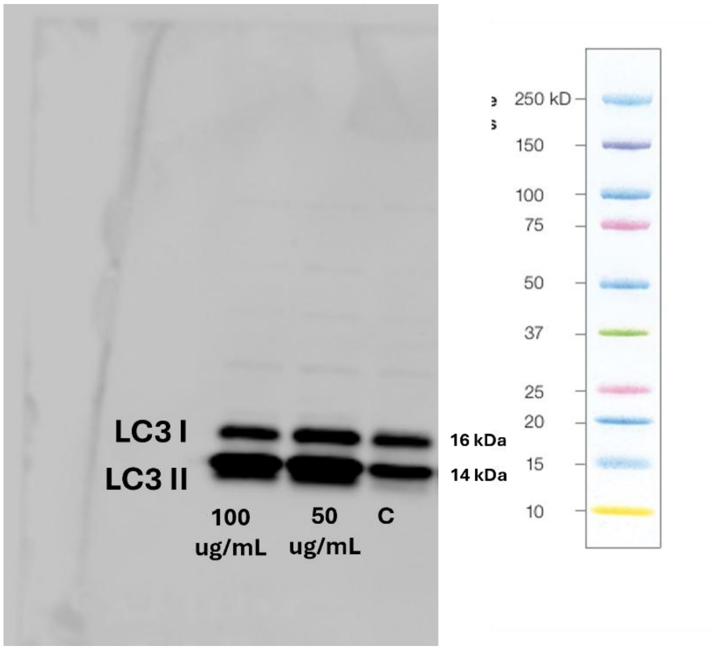

Figure 3A-LC3 I and II

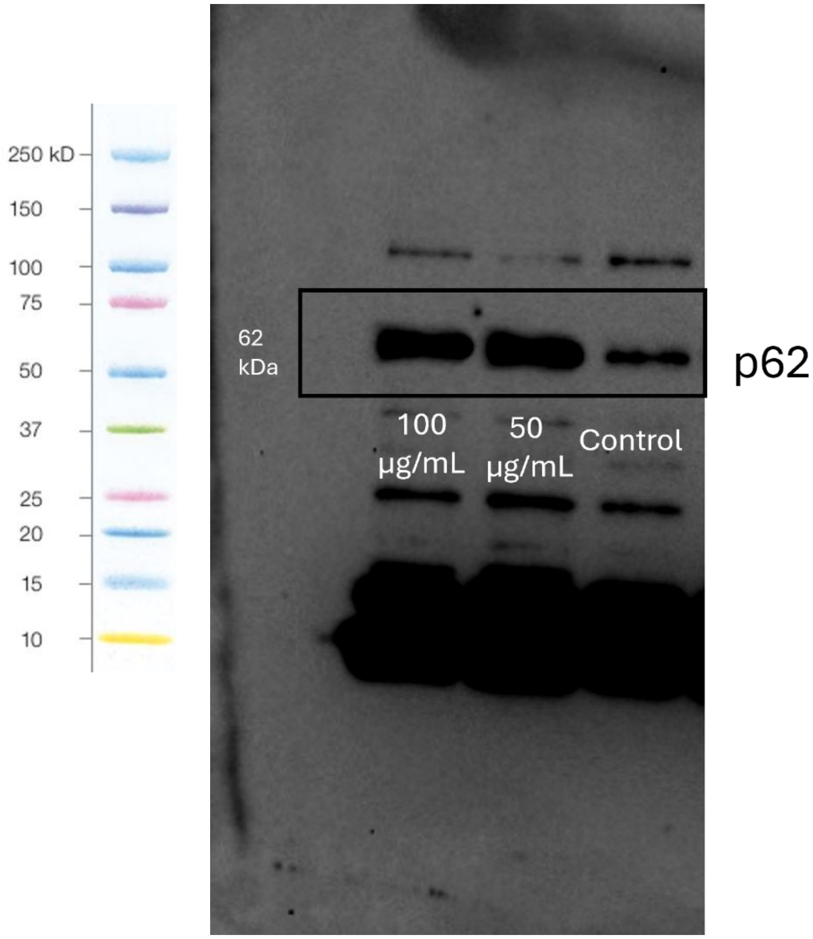

Figure 3A_p62

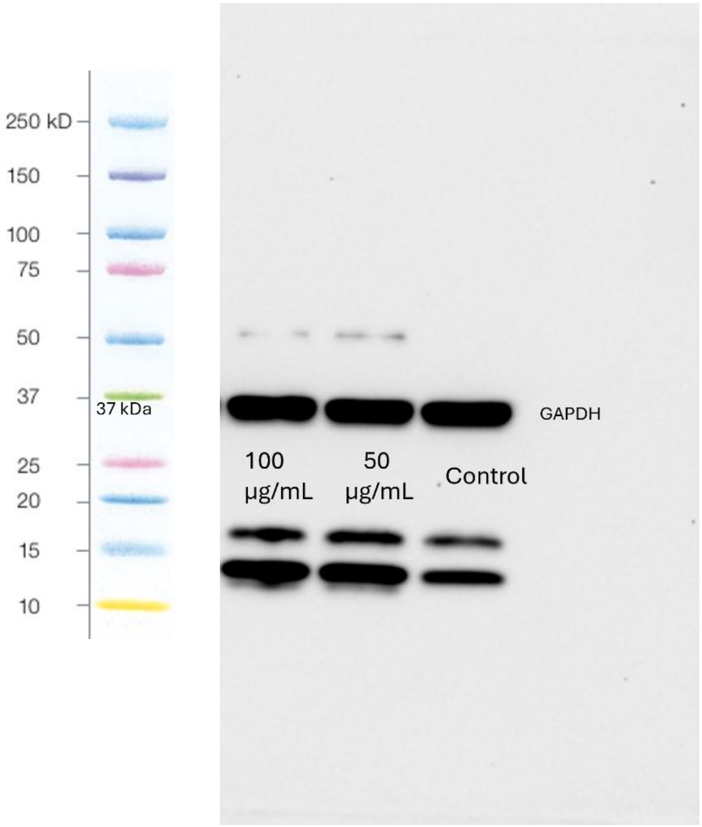

Figure 3A-GAPDH for autophagy protein series

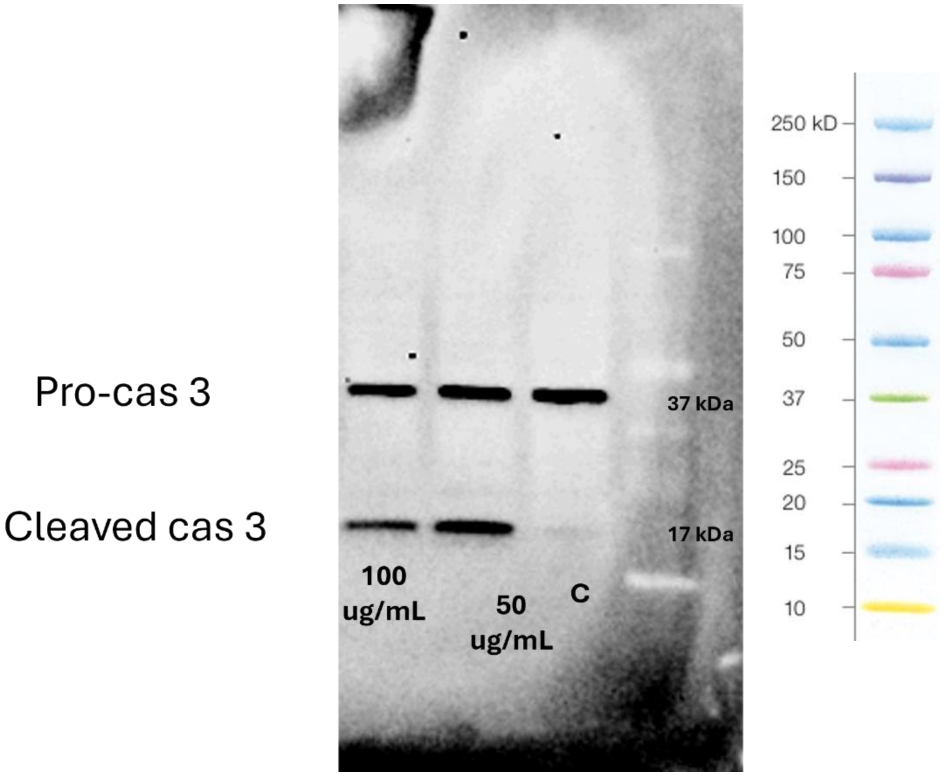

Figure 4A_ Caspase 3

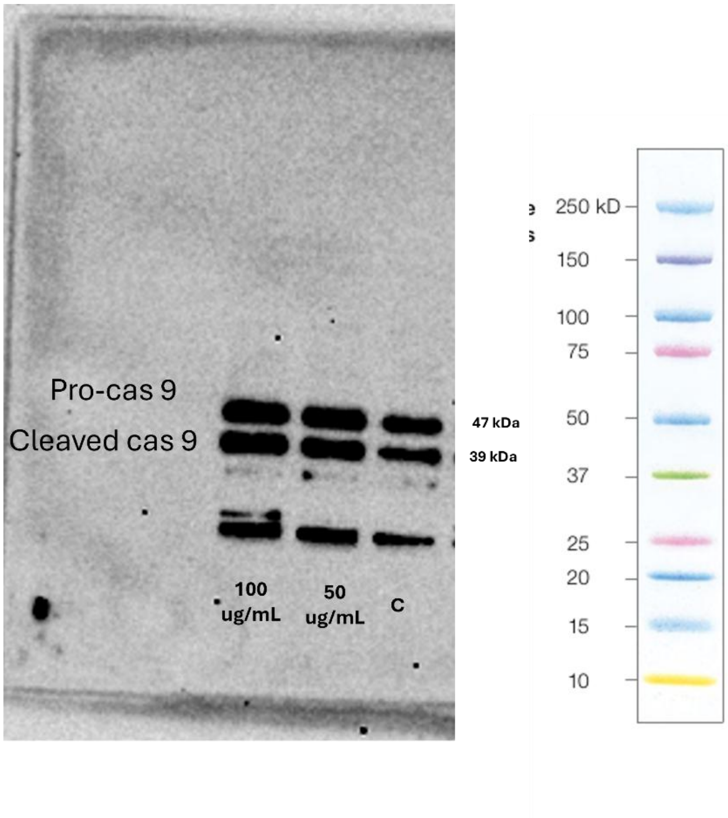

Figure 4A_ Caspase 9

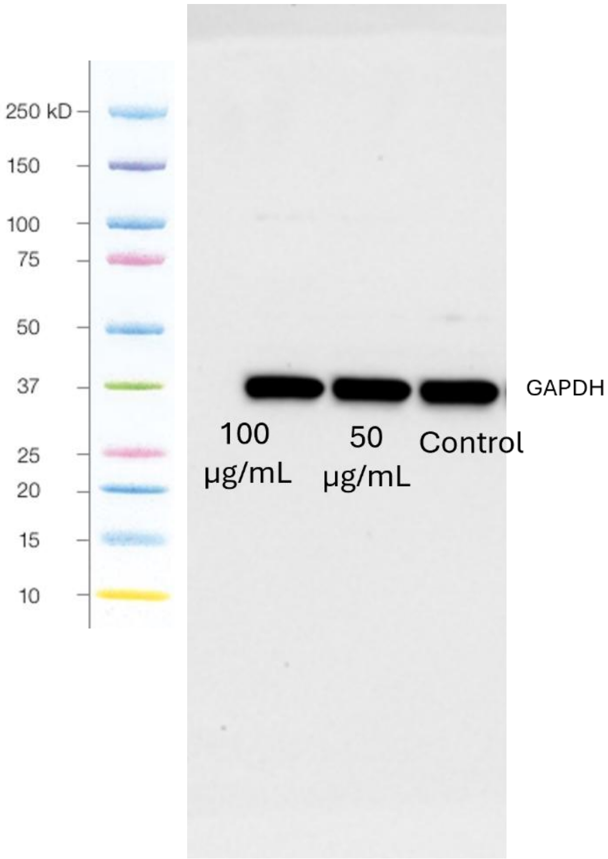

Figure 4A_GAPDH_for caspase series

Disclosure Regarding Molecular Weight Marker Images

The molecular weight marker images shown in this document were not acquired from the original chemiluminescent Western blot images. During the experiments, the molecular weight marker was not detectable under the chemiluminescent (ECL) imaging conditions employed for protein visualization and therefore was not captured in the original blot images.

For the purpose of molecular weight reference and annotation, a representative molecular weight marker image supplied by the manufacturer was included in this document. The same reference image has been used consistently across the annotated blot figures to indicate the approximate molecular weights of the detected proteins.

Accordingly, the molecular weight marker images presented herein are illustrative only and do not represent the actual chemiluminescent signal or the original marker appearance on each individual membrane. All protein bands, lane assignments, and experimental data shown in the raw blot images correspond to the original experimental records.
